# Research on urban country parks based on emergency medical facilities for public health emergencies—a case study of Guangzhou, China

**DOI:** 10.1007/s11356-023-28157-w

**Published:** 2023-06-13

**Authors:** Shuai Li, Zheng Wu, Jiefang Tang, Shuo Wang, Pengfei Wang

**Affiliations:** 1 College of Landscape Architecture and Art, Henan Agricultural University, Zhengzhou, 450002 China; 2 School of Public Policy & Management, China University of Mining and Technology, Xuzhou, 221000 China

**Keywords:** COVID-19, Public health emergency, Emergency medical facility, Country park, Site selection

## Abstract

As COVID-19 has swept across the world, the escalating number of confirmed and suspected cases overwhelmed the admission capacity of the designated hospitals. Faced with such a grim situation, governments made a quick decision to build emergency medical facilities to address the outbreak. However, the emergency medical facilities faced a huge risk of epidemic spread and improper site could lead to serious secondary transmission. Using the disaster prevention and risk avoidance function of urban green space can solve the problem of selecting the location of emergency medical facilities to a certain extent, with country parks having a high degree of compatibility with the latter. Based on the location requirements of emergency medical facilities, using Analytic Hierarchy Process and Delphi method, through analyzing the type of country parks, effective risk avoidance area, spatial fragmentation, distance from water sources, wind direction, and distance from the city, quantification of 8 impact factors such as hydrogeology and traffic duration was conducted to comprehensively compare 30 country parks in Guangzhou. The results showed that the overall quality of country parks approximated a normal distribution, with Lianma Forest Country Park having the highest comprehensive score and the most balanced distribution of scores for various impact factors. Considering safety, expandability, rehabilitation, convenience, pollution prevention, and fecal isolation, it is a preferred destination for emergency medical facility construction.

## Introduction

In December 2019, the COVID-19 broke out in Wuhan, China, and quickly spread around the world. The epidemic has spread to more than 200 countries and regions worldwide, becoming the longest lasting, most widespread, and most infected public health event since the 21st century. Globally, as of March 2023, the World Health Organization has reported 761,071,826 confirmed cases of COVID-19, including 6,879,677 deaths. COVID-19 is an infectious disease caused by SARS-CoV-2 coronavirus. Research has found that this virus has multiple transmission routes, including direct routes through air, wastewater, and surfaces (such as sneezing and coughing) (Wang and Liu 2021a, Zhai and Jiang 2020), and indirect transmission through aerosols, such as flushing toilets. SARS-CoV-2 virus has an incubation period, typically 1 to 14 days, mostly 3 to 7 days, and is infectious during the incubation period (Lauer et al. 2020, Zhai and Jiang 2020). This scale and speed of transmission has led to the virus being able to spread throughout the world in a short time, posing a significant challenge to public health security worldwide.

In the process of fighting against COVID-19, many countries have noticed the importance of emergency medical facilities. On the one hand, it can effectively control the spread of the epidemic and prevent the number of infected cases from soaring. On the other hand, it facilitates the isolation, treatment, and monitoring of confirmed patients, effectively relieving the pressure of receiving patients in designated hospitals and enabling critical patients to receive timely treatment. At the beginning of 2020, after the outbreak of COVID-19, Wuhan built *Huoshenshan* Hospital and *Leishenshan* Hospital to isolate and treat confirmed patients (Winch et al. 2021), referring to the model of “Beijing *Xiaotangshan* Hospital” during the fight against SARS in 2003 (AlTakarli 2020). After March 19, 2020, there were no new confirmed or suspected cases in Wuhan for 5 consecutive days, and the epidemic center was later transferred to Russia, Italy, Spain, Germany, the United States, and other countries (Liu et al. 2020a). Health systems in the United States and around the world have been disrupted by the COVID-19 pandemic (Baughman et al. 2020). In order to alleviate the shortage of medical resources, governments have quickly established emergency medical facilities. For example, the emergency hospital in New York City is built within Central Park (Central Park 2020). In Madrid, the exhibition center was transformed into a temporary hospital (Candel et al. 2021). In Mulhouse City, the hospital parking lot serves as the location of the field hospital (Platto et al. 2021).

**Fig. 1:**
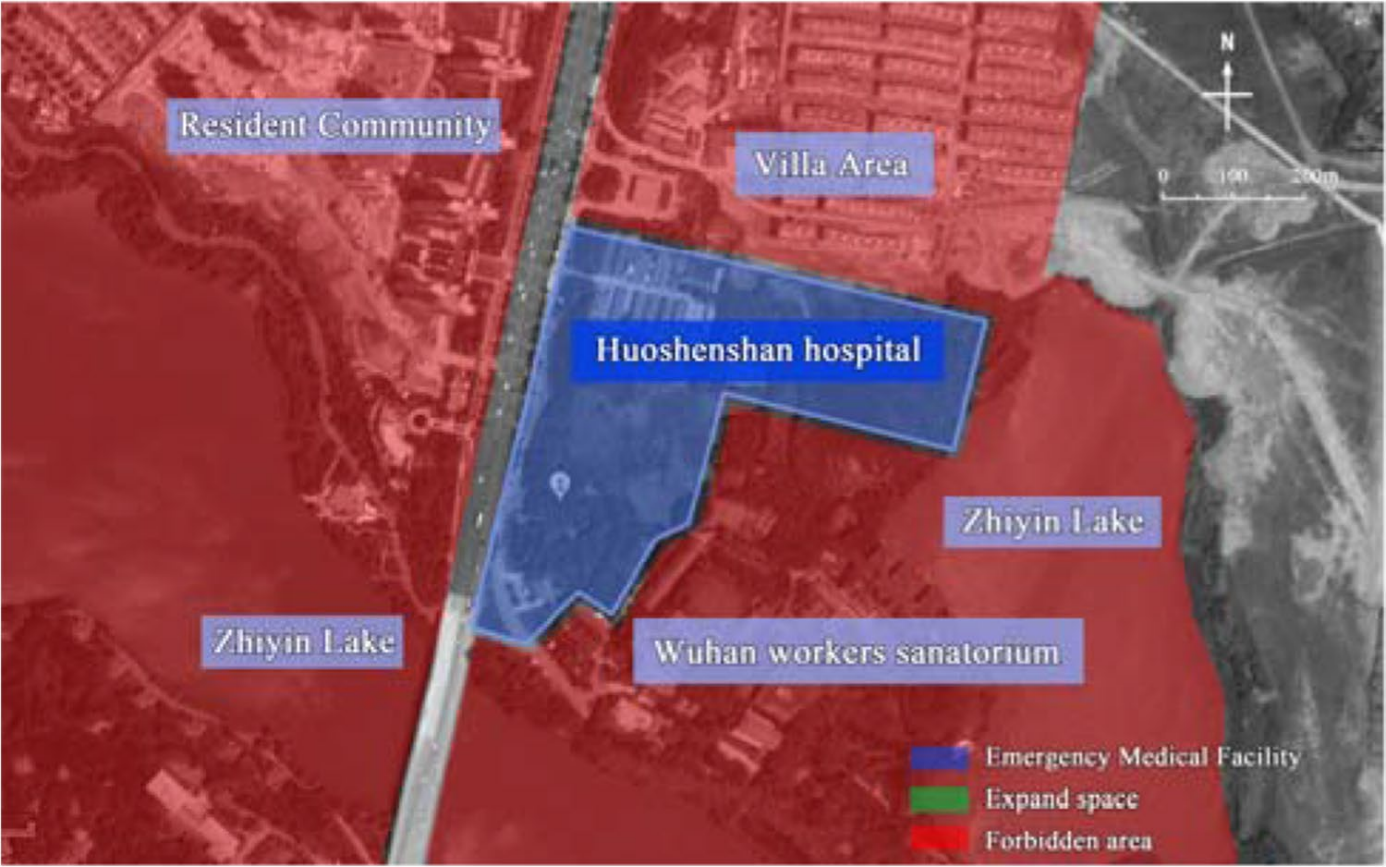
Site environment of Wuhan *Huoshenshan* Hospital

**Fig. 2:**
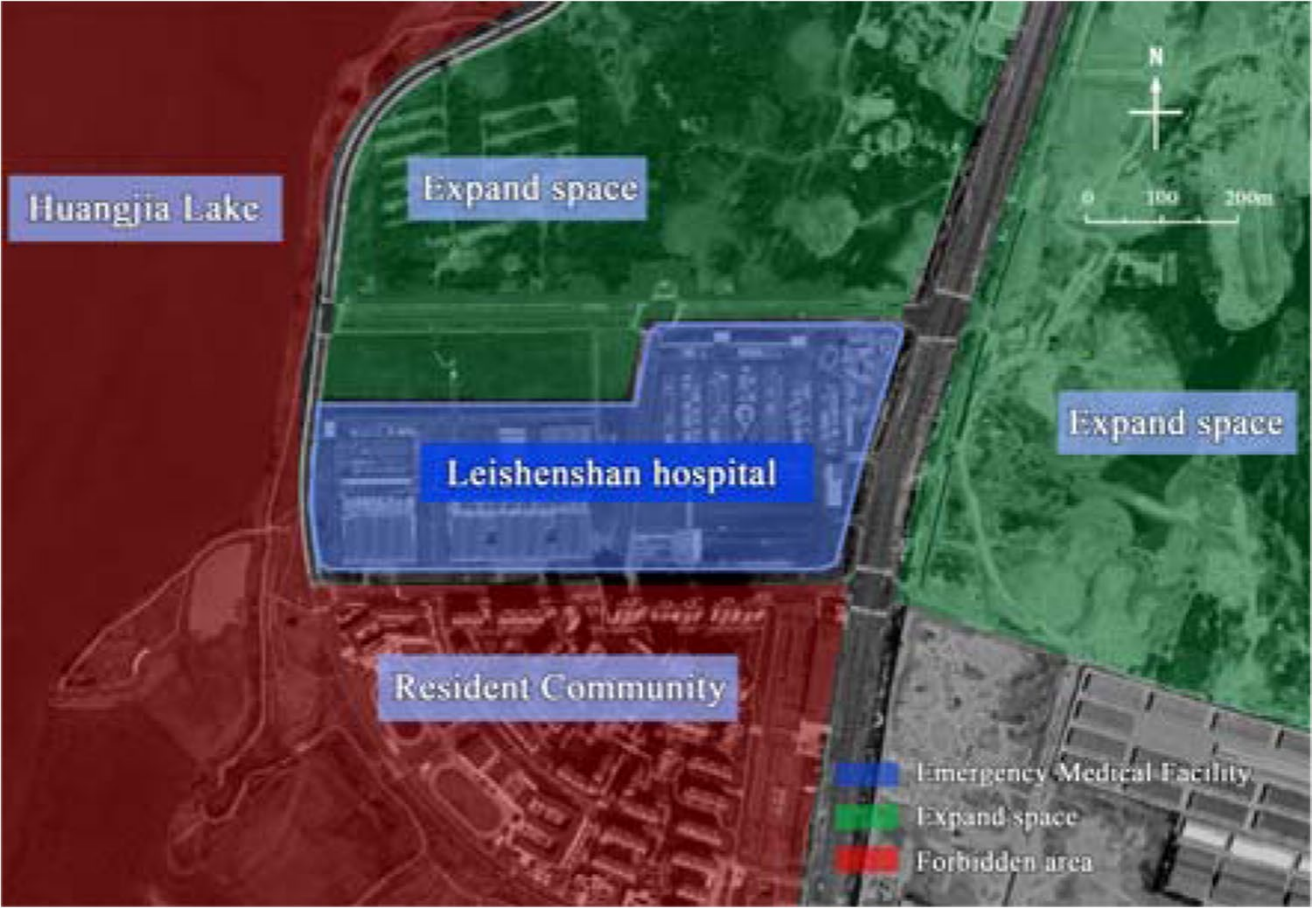
Site environment of Wuhan *Leishenshan* Hospital

Thus, the emergency medical facilities have been an important means to control the spread of the epidemic (Cao and Chen 2019), and the safety of site selection and the high efficiency of construction are the prerequisites and guarantees for emergency medical facilities to play the role of rescue (Tang et al. 2020). For example, the rapid completion of *Huoshenshan* Hospital and *Leishenshan* Hospital had bought valuable time for rescuing patients in Wuhan, China. Although *Huoshenshan* Hospital (Zhou et al. 2020) and *Leishenshan* Hospital have convenient traffic conditions and reasonable wind directions, the scientific nature of their site selection needs to be discussed (Figs. 1 and 2). In view of the requirements for treatment of infectious diseases, emergency medical facilities should have convenient transportation, remoteness, isolation, and other conditions. First of all, the two hospitals should not be built near the lake, which may provide a vector for water-borne infectious disease. Secondly, with the rapid growth of the number of patients, the *Huoshenshan* Hospital is surrounded by Wuhan Staff Sanatorium, Zhiyin Lake, and two residential areas (Fig. 1), without leaving enough space for secondary expansion. In contrast, the *Leishenshan* Hospital left a large amount of open space in both the east and north (Fig. 2). As the epidemic grew, *Leishenshan* Hospital (Tang et al. 2021) was successively expanded from the planned 50,000 m^2^ to 75,000 m^2^ and then to 79,900 m^2^ during the construction process, which not only saved time and materials but also provided convenience for centralized treatment and isolation.

Urban green space is an ideal refuge for residents to resist urban disasters. WHO mentioned in the Report on Urban Green Space and Health that urban green space can stimulate social cohesion, support sports activities, reduce exposure to air pollutants, noise, etc., promote the physical and mental health of urban residents, and reduce incidence rate and mortality by providing psychological relaxation and relieving pressure (World Health Organization 2016). With the outbreak of COVID-19, many scholars have paid attention to the impact of urban public space and urban green space on it. Lu Liu studied the spread of COVID-19 from the urban perspective and mentioned that urban planning with more available open space is essential for combating various disasters and disasters (Liu 2020). Xu Xian et al. sorted out the latest progress of research on the urban disaster-prevention green space (Xu and Zhang 2020b). Anson et al. focused on the positive role played by country parks during the outbreak (Ma et al. 2021). Tang Jiefang et al. suggested that suburban forest parks are highly compatible with the siting of emergency medical facilities (Tang et al. 2021). Li Shuai et al. established a quantitative evaluation system for the location of emergency medical facilities (Li et al. 2021). Ouyang Dong and others proposed to incorporate treatment facilities for public health emergencies into the national spatial planning system, and country parks can be used as temporary and dedicated treatment facilities for construction (Ouyang et al. 2020). Wang Jun and others discussed the preliminary design of emergency medical facilities in country parks (Wang et al. 2021b). Lu Xun et al. promoted the planning and construction of country park infectious disease hospitals to build urban epidemic prevention barriers (Lu et al. 2020). Li Liang et al. discussed the relationship between landscape architecture and public health in the post epidemic era and were concerned that the parks located on the edge of the urban have the advantages of low construction cost, sufficient space, and easy isolation. Based on the epidemic prevention and control, the author puts forward the reform strategy of infrastructures such as transportation, anti-seepage, and drainage (Li and Yang 2020).

In response to the above issues, by comparing factors such as safety, transportation conditions, land use scale, and infrastructure, it was found that the site selection requirements for country parks and emergency medical facilities have a high degree of compatibility. In terms of current development status, there are usually several or even dozens of country parks built around large cities, and there are significant differences in their scale, traffic conditions, relative location and distance from the main urban area, wind direction, and groundwater level. Only by quantifying and comparing the factors that affect the location of emergency medical facilities can the optimal items be further selected among many parks, and through the design method of combining disaster relief with disaster relief, the master planning that can be implemented for emergency medical facility plans can be provided before the epidemic.

## Research objects and methods

### Overview of the research site

**Fig. 3:**
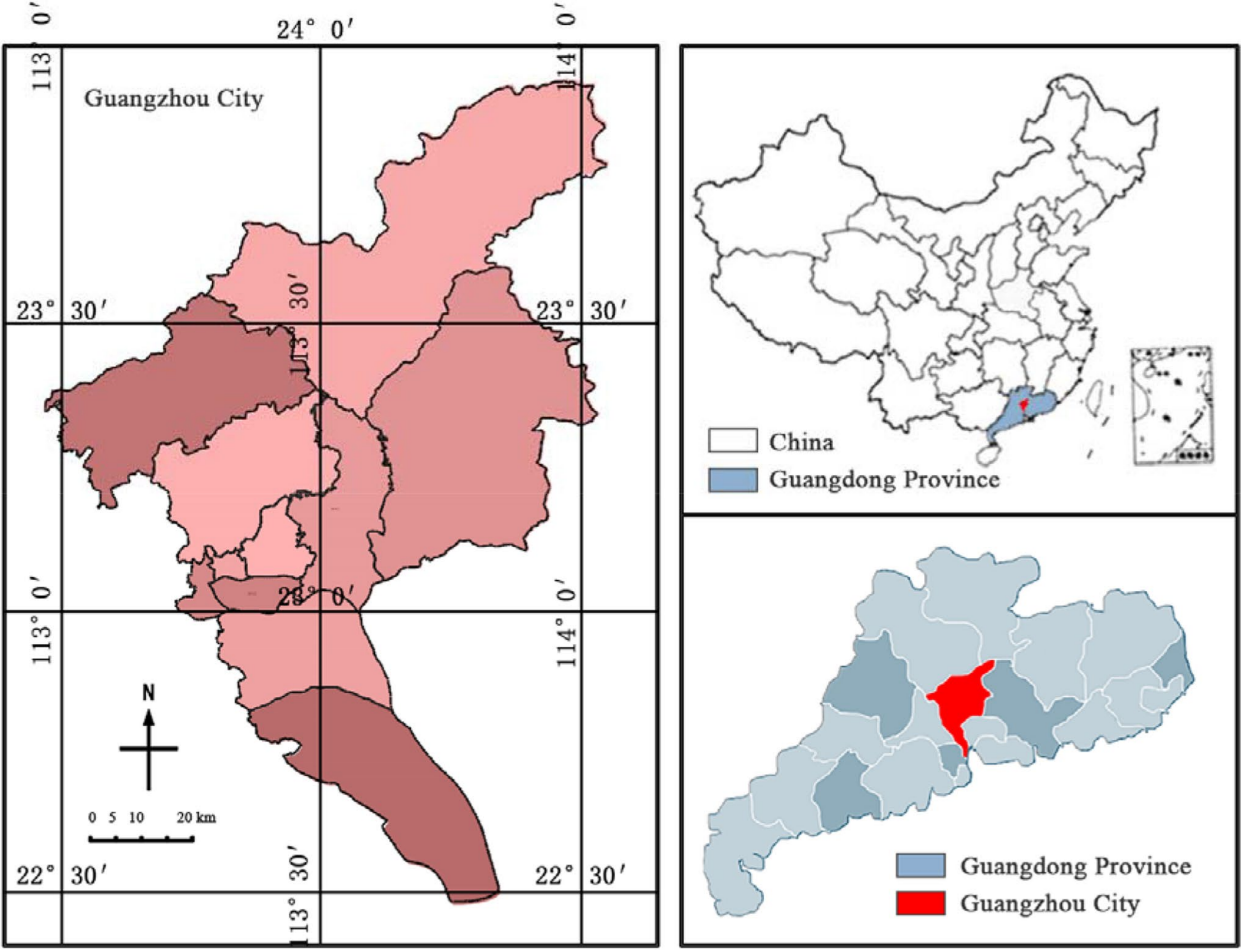
Location of Guangzhou City

At the beginning of 2020, the COVID-19 broke out in Wuhan, Hubei Province, China, and local medical institutions encountered tremendous pressure in a short period of time. The Wuhan Municipal Government immediately built *Huoshenshan* and *Leishenshan* Hospitals, increased the ability to treat patients with COVID-19, effectively alleviated the shortage of local hospital beds, and played a key role in combating the COVID-19. In May 2021, a new round of epidemic caused by the imported Delta variant virus strains from abroad broke out in Guangzhou (Zhang et al. 2021b). Compared with the COVID-19 in Wuhan, this round of epidemic in Guangzhou has stronger transmission capacity and faster transmission speed and is more likely to cause large-scale infection. Located in the south of Chinese Mainland, Guangzhou is China’s “southern gate” to the world. It has a developed transportation network and a large number of entry-exit population. Improper prevention and control can easily cause the spread of imported epidemic diseases abroad. Therefore, it is imperative to draw on the experience of establishing emergency medical hospitals (*Huoshenshan* Hospital and *Leishenshan* Hospital) in Wuhan and develop corresponding emergency plans in Guangzhou (Fig. 3).

Guangzhou is located in the south of Chinese Mainland, on the northern edge of the Pearl River Delta, near the South China Sea, adjacent to Hong Kong and Macao. It is a regional central city, transportation and communication hub in South China. As the birthplace of the “Maritime Silk Road” in ancient China, Guangzhou has been an important port for China’s foreign trade since more than 2200 years ago, known as the “millennium commercial capital.” By the end of 2019 (Guangzhou Statistics Bureau 2021), the permanent population of the city was 15.3559 million, with a permanent population density of 2059 people/km^2^. Guangzhou is the intersection of the Beijing-Guangzhou, Guangzhou-Shenzhen, Guangzhou-Maoming, and Guangzhou-Meizhou-Shantou railways and the civil aviation transportation center of South China, with extremely close connections throughout the country. In 2019, the annual passenger traffic volume reached 498.19 million person-times, ranking first in China in terms of regional GDP. The huge population size and high frequency mobility are the key to the rapid spread of the epidemic (Liu et al. 2020b).

### Research objects and data sources

**Fig. 4:**
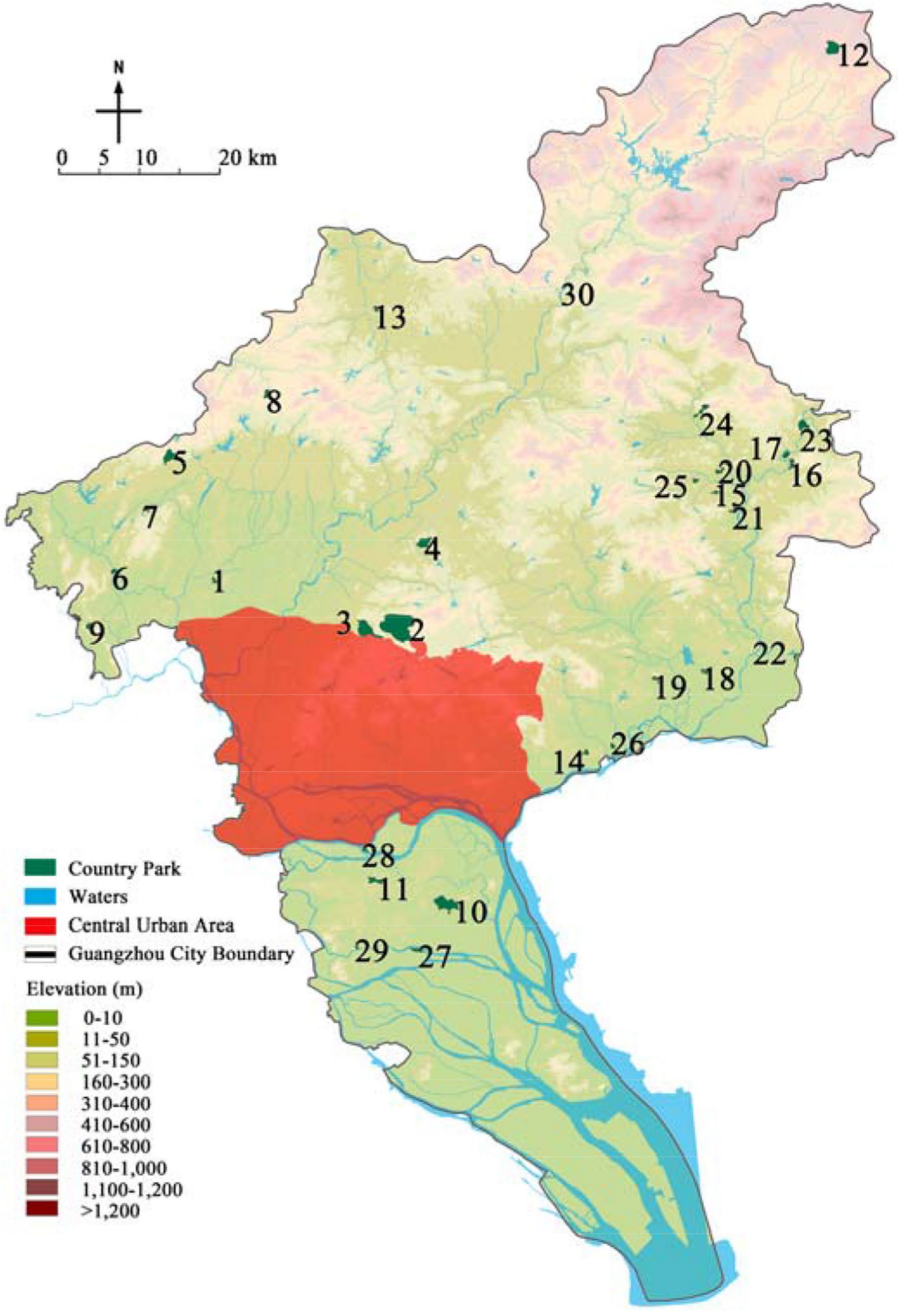
Distribution of research country parks

This paper took Guangzhou country parks as the research object and Guangzhou administrative boundary as the research boundary, covering an area of 7434.4 km^2^. Based on the list in the Guangzhou City Greenland System Plan (2020–2035), after screening, a total of 30 parks were finally determined as the research objects (Table 1 and Fig. 4).

**Table 1:** List of research objects

Park number	Park name	Park number	Park name	Park number	Park name	Park number	Park name
1	Daling Forest Park	9	Pinglingtou Forest Park	17	Menghuabu Forest Park	25	Labu Forest Park
2	Baishan Forest Park	10	Jianfengshan Forest Park	18	Shitanmache Forest Park	26	Dadun Forest Park
3	Helong Forest Park	11	Dazhengang Forest Park	19	Wudieling Forest Park	27	Caohe Wetland Park
4	Madong Forest Park	12	Lianma Forest Park	20	Dongxijing Forest Park	28	Wanjutou Wetland Park
5	Panguwang Forest Park	13	Maling Forest Park	21	Hexiangu Forest Park	29	Lianhuyong Wetland Park
6	Hualing Forest Park	14	Siwanggang Forest Park	22	Dapuwei Forest Park	30	Conghua Hot Spring Wetland Park
7	Yishan Forest Park	15	Houlongshan Forest Park	23	Xihutan Forest Park		
8	Yanyinghu Forest Park	16	Jiufengshan Forest Park	24	Shilongtou Forest Park

Firstly, in accordance with the requirements of “The Design Guidelines for Hospitals for Patients with SARS” (National Health Commission of the People’s Republic of China 2004) and “The Design Standards of Emergency Medical Facilities for COVID-19” (China Association for Engineering Construction Standardization 2020), combined with the site selection characteristics of emergency medical facilities and the design principles of infectious hospitals, eight impact factors including park type, effective avoidance area, spatial fragmentation degree, water source protection area, wind direction, distance from city center, impermeability, and transport duration of country parks were set as impact factors.

Park type (*f1*): country parks are divided into different categories according to their landscape characteristics, surface morphology, and resource types.

Effective avoidance area (*f2*): the effective refuge area of the country park refers to the total area of the park minus the water area, traffic, buildings and their falling objects, the area affected by collapse, and other areas, which can be used for the construction of emergency medical facilities.

Spatial fragmentation degree (*f3*): spatial patch is the most common form of landscape pattern, which reflects the heterogeneity of landscape, and spatial fragmentation degree reflects the complexity of park spatial structure (Xu et al. 2020a).

Water source protection area (*f4*): a certain area designated to prevent water source pollution and ensure water quality and requires special protection.

Wind direction (*f5*): the direction of wind in the city. Since the dominant wind direction in the city is not obvious, the impact of wind direction on site selection is expressed by quantifying the wind frequency in all directions.

Distance from the city center (*f6*): the distance from each country park to the city center.

Impermeability (*f7*): the buried depth of groundwater refers to the buried depth of groundwater, that is, the distance from the groundwater surface to the ground. Permeability coefficient is a quantitative indicator to measure the permeability of groundwater in the site. The ratio of groundwater depth to permeability coefficient can be used as an indicator to measure the degree of water resistance in the target area.

Transport duration (*f8*): the time required to transfer COVID-19 patients from designated hospitals in the urban area to emergency medical facilities.

Secondly, Delphi method and Analytic Hierarchy Process (AHP) assign weights for each impact factor (Zhang et al. 2021a), with the listing of each required impact factor and the collection of expert opinions by e-mail and summarizing the more concentrated opinions put forward by experts after multiple rounds of feedback (Table 2).

**Table 2:** Weight of impact factors

Number	1	2	3	4	5	6	7	8	—
Impact factor (*f*_*i*_)	Park type	Effective avoidance area	Spatial fragmentation degree	Water source protection area	Wind direction	Distance from the city center	Impermeability	Transport duration	Total
Weight (*w*_*i*_)	16	12	20	24	12	8	4	4	100

Thirdly, each impact factor was graded using a five-tier system, in order of I, II, III, IV, and V (*f*_*i*_) from inferior to superior. Multiply the grade (*f*_*i*_) by the corresponding weight (*w*_*i*_) to obtain the impact factor score (*y*_*i*_) in Eqs. 1 and 2, and then, use Eqs. 3 and 4 to add the scores of each impact factor to obtain the comprehensive score (*Y*) of the country park (Zhang and Wu 2023).

1 $$\sum_{i=1}^8{w}_i=100$$

2 $${y}_i={w}_i{f}_i$$

3 $$Y=\sum_{i=1}^8{w}_i{f}_i$$

4 $$Y=16{f}_1+12{f}_2+20{f}_3+24{f}_4+12{f}_5+8{f}_6+4{f}_7+4{f}_8$$

Fourth, the comprehensive scores of 30 country parks were calculated and ranked. Parks with higher scores are more suitable as the site selection of emergency medical facilities than those with lower scores.

## Impact factor assignment method

### Park type (*f*_*1*_)

The 30 country parks in “The Guangzhou City Greenland System Plan (2020–2035)” could be divided into five types: wetland, heritage, mountain, pastoral, and forest. The water polluted pathogenic microorganisms are an important vector of diseases, for instance, cholera, hepatitis A, and typhoid fever can be transmitted by water. Among the 37 legal infectious diseases in China, 8 are water-borne infectious diseases (Lv et al. 2017). Wetland country parks are close to water sources and are vulnerable if they are not properly protected. Therefore, wetland country parks were set as grade I. In addition, more than 100 zoonotic infectious diseases spread rapidly between people and animals, so zoos should not be used as the site of emergency medical facilities. For example, the Wanzuitou Wetland Park is the urban wildlife demonstration site, so it was also set as grade I. The historical landscape is a non-renewable resource, so the heritage parks were set as grade II. Guangzhou is one of the first batches of famous historical and cultural cities in China, with many cultural relics above and below ground, such as Panguwang Forest Park and Hexiangu Forest Park, which were all heritage parks. Considering uncertainties such as traffic and geological conditions, the mountain parks were set as grade III. The pastoral parks have flat terrain and relatively complete infrastructure, so they were set as grade IV. The forest parks have excellent ecological environment, with large tracts of trees in the park playing a role in isolation, so they were set as grade V (Zhang et al. 2019a).

### Effective avoidance area (*f*_*2*_)

The construction site of emergency medical facilities should consider the lower limit of its scale. According to the number of casualties in the first-level public health emergency and the scale of the existing facilities (Table 3), it could be known that when the emergency medical facility was a single-story building, the area should not be less than 4.7 hm^2^ (Tang et al. 2020). Statistics showed that the available construction area of these country parks ranged from 7.06 to 979.38 hm^2^, all of which met the site requirement. The parks below 25 hm^2^ were grade I, 25–50 hm^2^ were grade II, 50–75 hm^2^ were grade III, 75–100 hm^2^ were grade IV, and above 100 hm^2^ were grade V.

**Table 3:** Scale of existing emergency medical facilities and number of beds

Emergency medical facility	Scale (m^2^)	Beds	A bed (m^2^)
Xiaotangshan Hospital	40,000	1000	40
Huoshenshan Hospital	50,000	1000	50
Leishenshan Hospital	80,000	1600	50

### Spatial fragmentation degree (*f*_*3*_)

Emergency medical facilities required a single, homogeneous, and continuous construction site. Therefore, parks with few patches and low landscape fragmentation are more suitable as emergency medical facility construction sites. Landscape fragmentation degree is expressed by the ratio of the number of patches to the corresponding area, that is, *C*_*i*_ = *N*_*i*_/*A*_*i*_ (Zhang and Zhang 2019b). The greater the degree of fragmentation, the worse the integration of the park space, which is not conducive to the construction of emergency medical facilities. Statistics showed that the spatial fragmentation degree of 30 country parks were between 0.0046 and 0.8772. According to the distribution characteristics of fragmentation degree, the parks were grade V below 0.02, grade IV between 0.02 and 0.04, grade III between 0.04 and 0.06, grade II between 0.06 and 0.1, and grade I above 0.1.

### Water source protection area (*f*_*4*_)

Guangzhou has a well-developed water system with numerous rivers, lakes, and reservoirs. The total water area is 744 km^2^, accounting for 10.05% of the city’s area. In the “Technical guideline for delineating source water protection areas,” the land area protection scope of different types of water sources is delimited, respectively, of which large reservoirs are the most stringent. For river type water source, the land area protection scope of first class and second class is limited by the coastal horizontal distance of 50 m and 1000 m, respectively. For lakes and reservoirs, primary protection includes a 200 m land area above the normal water level on the side of the water intake; in secondary protection, depending on the scale, it is divided into two classification methods: “no less than 3000 m outside the primary protection area” and “2000 m above the normal water level” (Ministry of Ecology And Environment of the People’s Republic of China 2018). According to the characteristics of water source types in Guangzhou and the principle of strict division, the above division boundaries were integrated. The land area within 200 m (including water source) was set as grade I, 200–500 m as grade II, 500–1000 m as grade III, 1000–2000 m as grade IV, and more than 2000 m as grade V.

### Wind direction (*f*_*5*_)

Emergency medical facilities should be located in the downwind area of the city. Because the dominant wind direction in some cities is not obvious or there are more than two dominant wind directions, the 16 compass point method could be used to quantify the wind frequency in all directions. According to the statistics of meteorological data in Guangzhou, the frequencies of 16 wind directions are shown in Table 4. All wind frequencies could be divided into five bands: 12%–15%, 9%–12%, 6%–9%, 3%–6%, and 0–3%, corresponding to parks in the order of grade I, II, III, IV, and V.

**Table 4:** Annual frequency of wind directions in Guangzhou (%)

Wind direction	N	NNE	NE	ENE	E	ESE	SE	SSE	C
Frequency	13.4	4.26	6.44	3.96	11.73	6.7	13.02	7.23	
Wind direction	S	SSW	SW	WSW	W	WNW	NW	NNW	–
Frequency	7.64	4.56	3.63	2.2	2.73	2.6	4.66	5.24	–

### Distance from the city center (*f*_*6*_)

“Avoiding densely populated urban areas and environmentally sensitive areas” is an important principle for the location of emergency medical facilities, so the park should be far away from the urban area. Taking the administrative boundary of the central urban area of Guangzhou as the starting boundary, from near to far, it was set as grade I for 0–10 km, grade II for 10–20 km, grade III for 20–30 km, grade IV for 30–40 km, and grade V for more than 50 km. Since some cities have more than one central urban area, when the park was located in two grade regions at the same time, the principle of taking the value from the low was adopted.

### Impermeability (*f*_*7*_)

Medical waste carries a large number of pathogens, heavy metals, and organic pollutants, which can produce a variety of harmful leachate after rainwater and biological hydrolysis (National Health Commission of the People’s Republic of China 2013). In order to prevent harmful leachate from entering the soil with rainwater and polluting groundwater, emergency medical facilities are built in sites with high terrain, low groundwater level, and good water-resisting property. The isolation zone between the stratum surface and groundwater is called “aeration zone.” If the water-resisting property of aeration zone is good, the infiltration coefficient (*α*) is small. According to the geological conditions of Guangzhou, it could be divided into five areas such as *α*≤0.100, 0.100<*α*≤0.200, 0.200<*α*≤0.300, 0.300<*α*≤0.400, and *α*>0.400 (Liu et al. 2019), and the parks located in the corresponding areas were set as grade V, grade IV, grade III, grade II, and grade I, respectively.

### Transport duration (*f*_*8*_)

During the outbreak of the epidemic, a large number of critically ill patients need to be transferred from the designated hospital in the urban area to the location of emergency medical facilities through “negative pressure ambulance,” so the convenience of transportation is very important. In the same time period, through the digital map navigation system, using the driving mode, the time taken from the five designated hospitals (Health Commission of Guangdong Province 2021) to each country park was measured, and the longest transport duration was used for evaluation. According to the statistics, the longest driving time of the 30 country parks was 2.2 hours, and the traffic duration was divided into five periods: more than 120 min, 100–120 min, 80–100 min, 60–80 min, and less than 60 min, which were grade I, II, III, IV, and V in turn.

## Impact factor evaluation and analysis

**Fig. 5:**
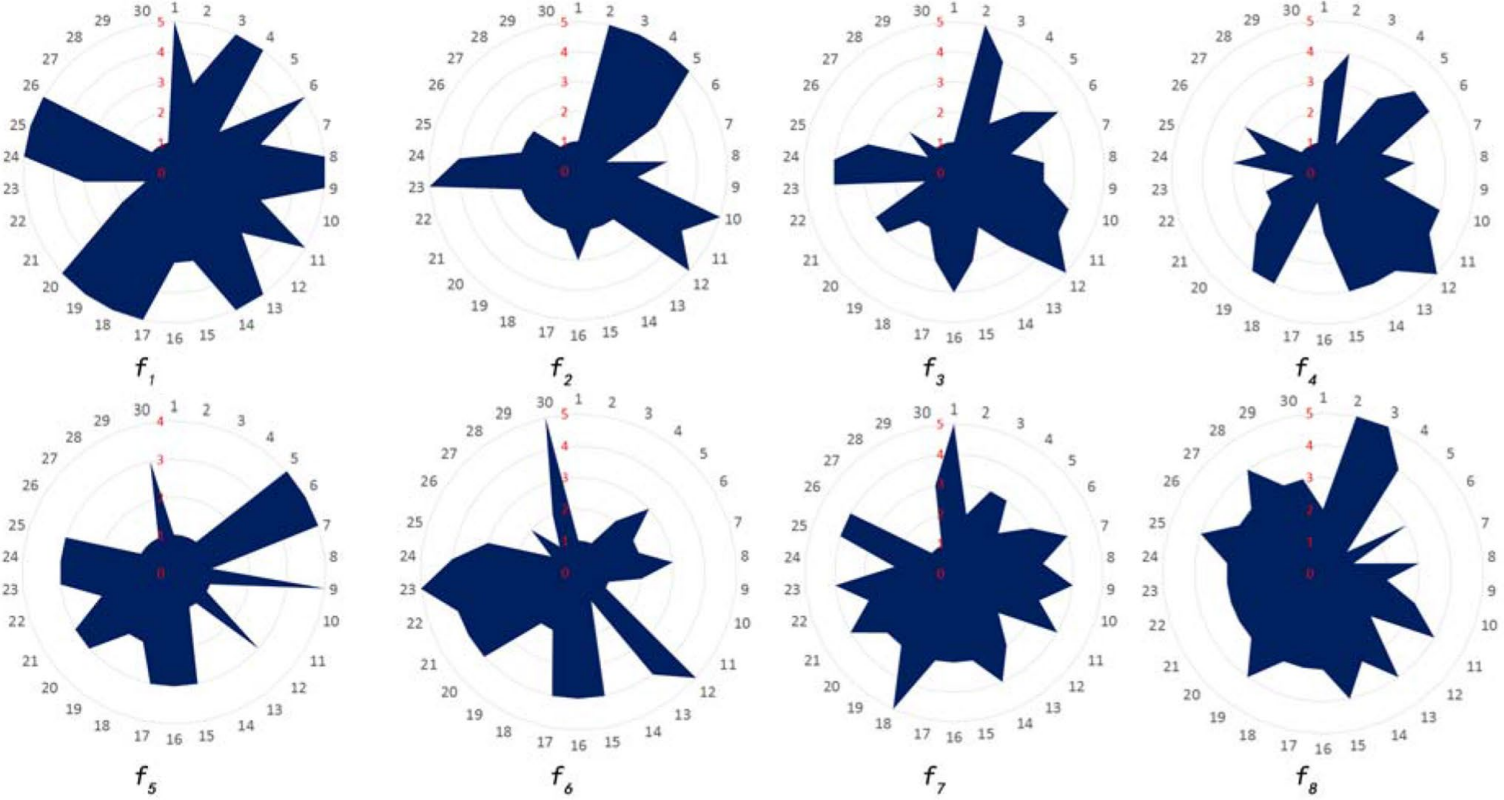
Scores of the impact factors

Based on the above assignment method, the impact factors of 30 country parks were assigned respectively (Table 5). The results showed that park type, water-resisting property, and transport duration were superior. The spatial fragmentation degree, the distance from waters, and the distance from urban were moderate. The available construction area and the wind direction were poor (Fig. 5).

**Table 5:** Data of the impact factors

Park number	Park type	Effective avoidance area (hm^2^)	Spatial fragmentation degree	Water source protection area (m)	Wind direction	Distance from the city center (km)	Impermeability (*α*)	Transport duration (h)
1	Forest park	24.74	0.1617	500–1000	N	5.0	*α*≤0.100	1.7
2	Forest & mountain park	979.38	0.0061	1000–2000	N	3.8	0.300<*α*≤0.400	1
3	Forest park	179.63	0.0111	≤200	N	2.1	0.200<*α*≤0.300	0.94
4	Forest park	128.67	0.0466	500–1000	N	14	0.200<*α*≤0.300	1.2
5	Forest & heritage park	171.75	0.0233	1000–2000	NW	22	0.300<*α*≤0.400	2.2
6	Forest park	69.6	0.0144	1000–2000	NW	11.2	0.200<*α*≤0.300	1.6
7	Forest & mountain park	15.3	0.0654	200–500	NW	15.5	0.100<*α*≤0.200	2.1
8	Forest park	73.01	0.0274	500–1000	N	28.2	0.200<*α*≤0.300	1.5
9	Forest park	33.4	0.0299	200–500	NW	10.5	0.100<*α*≤0.200	1.7
10	Forest & mountain park	300.6	0.0200	1000–2000	N	10	0.200<*α*≤0.300	1.4
11	Forest park	95.33	0.0105	1000–2000	SE	5.2	0.100<*α*≤0.200	1.3
12	Forest & mountain park	215.7	0.0139	>2000	NE	91	0.300<*α*≤0.400	2
13	Forest park	34	0.0294	1000–2000	N	40	0.200<*α*≤0.300	1.3
14	Forest park	42.63	0.0469	1000–2000	N	7.7	0.100<*α*≤0.200	1.5
15	Forest & mountain park	32.5	0.0308	1000–2000	NE	31	0.200<*α*≤0.300	1.3
16	Forest & mountain park	58	0.0172	200–500	NE	38	0.200<*α*≤0.300	1.4
17	Forest park	40	0.0250	≤200	NE	39	0.200<*α*≤0.300	1.5
18	Forest park	38.8	0.0515	1000–2000	E	20	*α*≤0.100	1.4
19	Forest park	28.63	0.0699	1000–2000	E	15	0.200<*α*≤0.300	1.3
20	Forest park	31	0.0323	500–1000	NE	32	0.200<*α*≤0.300	1.4
21	Forest & heritage park	40.6	0.0246	200–500	NE	30.5	0.100<*α*≤0.200	1.4
22	Wetland park	27.38	0.1096	200–500	E	32	0.200<*α*≤0.300	1.6
23	Forest & mountain park	117.04	0.0171	≤200	NE	42	0.100<*α*≤0.200	1.6
24	Forest park	87.7	0.0114	500–1000	NE	36	0.300<*α*≤0.400	1.5
25	Forest park	30	0.0333	200–500	NE	29.5	0.100<*α*≤0.200	1.3
26	Forest park	27.36	0.1096	500–1000	N	10	0.100<*α*≤0.200	1.5
27	Wetland park	38.39	0.0521	≤200	N	13	*α*>0.400	1.4
28	Wetland park	15.82	0.1896	≤200	N	0.8	*α*>0.400	1.1
29	Wetland park	19.02	0.1052	≤200	N	12	*α*>0.400	1.4
30	Wetland park	7.06	0.7082	≤200	NE	48	0.200<*α*≤0.300	1.4

### Park type evaluation

Statistics on the types of the 30 parks (Table 6) showed that there were 5 in grade I, 2 in grade II, 7 in grade III, 0 in grade IV, and 16 in grade V. On the one hand, some country parks were upgraded from town-level forest parks, so the number of forest parks accounted for 53.33% of the total. On the other hand, some forest parks had other attributes, such as Jiufengshan Forest Park, which had overlapping mountains and great vertical fluctuation of terrain. In view of the principle of strict division, it was classified as mountain type. Overall, the park type was a favorable factor in the site selection process.

**Table 6:** Assignment results of the park types

Park number	1	2	3	4	5	6	7	8	9	10	11	12	13	14	15
Park type assignment	V	III	V	V	II	V	III	V	V	III	V	III	V	V	III
Park number	16	17	18	19	20	21	22	23	24	25	26	27	28	29	30
Park type assignment	III	V	V	V	V	II	I	III	V	V	V	I	I	I	I

### Effective avoidance area evaluation

According to the statistics of the effective avoidance area of the parks (Table 7), 5 of them were grade I, 13 were grade II, 3 were grade III, 2 were grade IV, and 7 were grade V. In general, the country parks were medium-sized, which was directly related to the location and level. In particular, it should be noted that the effective avoidance area of Conghua Hot Spring Wetland Park was only 7.06 hm^2^. When the epidemic situation increases rapidly, the park cannot provide sufficient space for the expansion of emergency medical facilities and should be selected carefully. In addition, in the selection process, the effective avoidance area should be comprehensively considered in conjunction with the spatial fragmentation degree.

**Table 7:** Assignment results of the effective avoidance area

Park number	1	2	3	4	5	6	7	8	9	10	11	12	13	14	15
Effective avoidance area assignment	I	V	V	V	V	III	I	III	II	V	IV	V	II	II	II
Park number	16	17	18	19	20	21	22	23	24	25	26	27	28	29	30
Effective avoidance area assignment	III	II	II	II	II	II	II	V	IV	II	II	II	I	I	I

### Spatial fragmentation degree evaluation

**Fig. 6:**
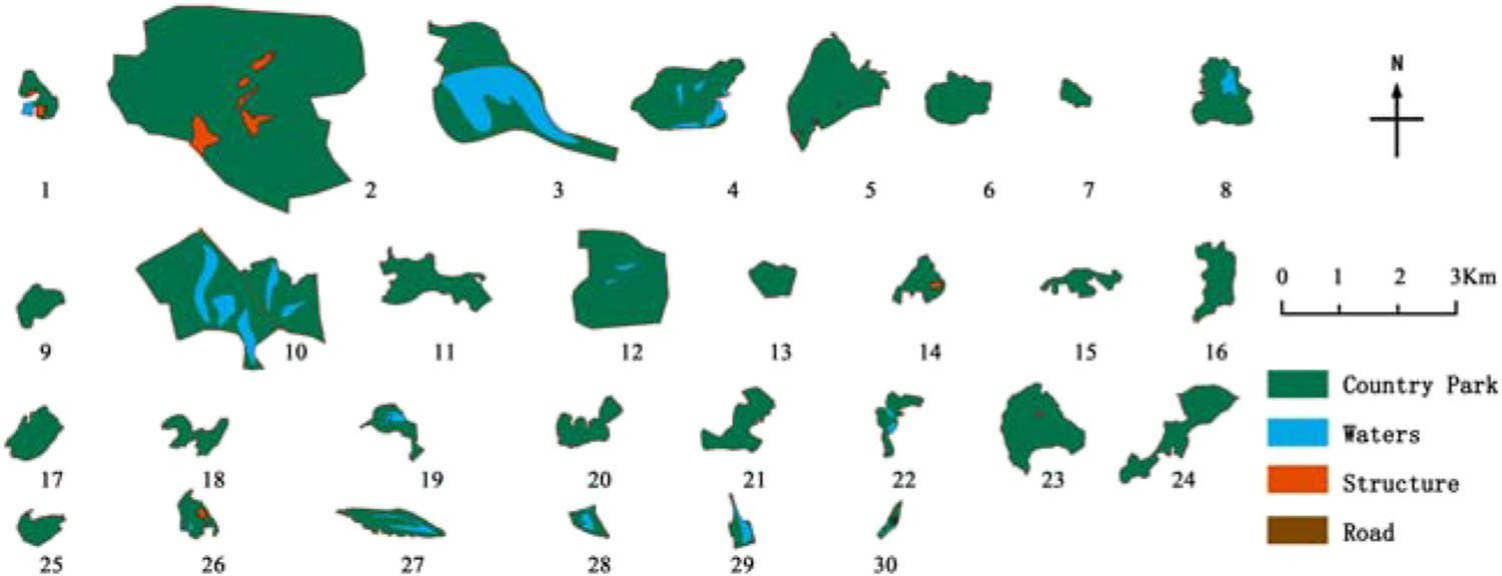
Spatial fragmentation degree of the country parks

According to the statistics of the spatial fragmentation degree (Table 8 and Fig. 6), there were 6 country parks in grade I area, 6 in grade II area, 9 in grade III area, 7 in grade IV area, and 2 in grade V. The comparison showed that the level of spatial fragmentation degree was highly consistent with the park scale.

**Table 8:** Assignment results of the spatial fragmentation degree

Park number	1	2	3	4	5	6	7	8	9	10	11	12	13	14	15
Spatial fragmentation degree assignment	I	V	IV	II	III	IV	II	III	III	IV	IV	V	III	II	III
Park number	16	17	18	19	20	21	22	23	24	25	26	27	28	29	30
Spatial fragmentation degree assignment	IV	III	II	II	III	III	I	IV	IV	III	I	II	I	I	I

### Water source protection area evaluation

**Fig. 7:**
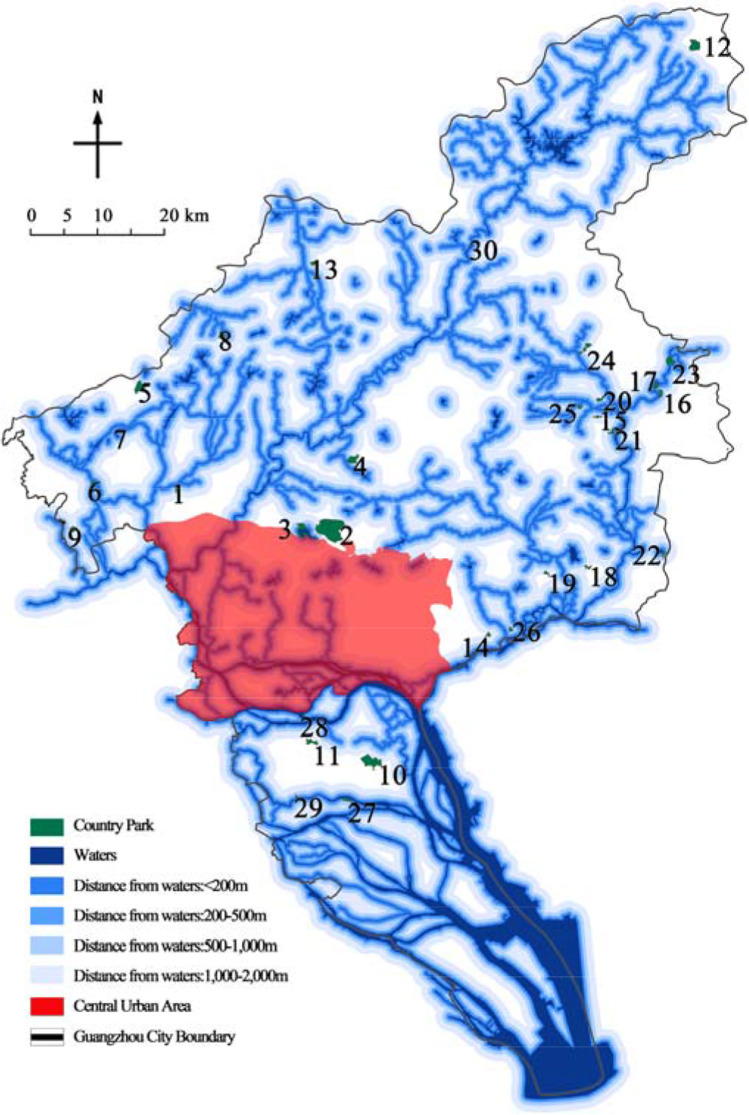
Water source protection area of the country parks

According to the statistics of the water source protection area (Table 9 and Fig. 7), there were 7 parks in grade I area, 6 in grade II area, 6 in grade III area, 10 in grade IV area, and 1 in grade V area. Among all the impact factors, the water source protection area had the highest weight, so it had the most significant impact on the comprehensive score of the parks. Taking the Xihutan Forest Park as an example, it had the outstanding advantages of effective avoidance area (117 hm^2^), long distance from the urban (40 km), low fragmentation (0.0171), and low permeability coefficient. However, the park is built near the Zengjiang River and is less than 200 m away from the waters, which greatly reduced its comprehensive score and ranking.

**Table 9:** Assignment results of the water source protection area

Park number	1	2	3	4	5	6	7	8	9	10	11	12	13	14	15
Water source protection area assignment	III	IV	I	III	IV	IV	II	III	II	IV	IV	V	IV	IV	IV
Park number	16	17	18	19	20	21	22	23	24	25	26	27	28	29	30
Water source protection area assignment	II	I	IV	IV	III	II	II	I	III	II	III	I	I	I	I

### Wind direction evaluation

**Fig. 8:**
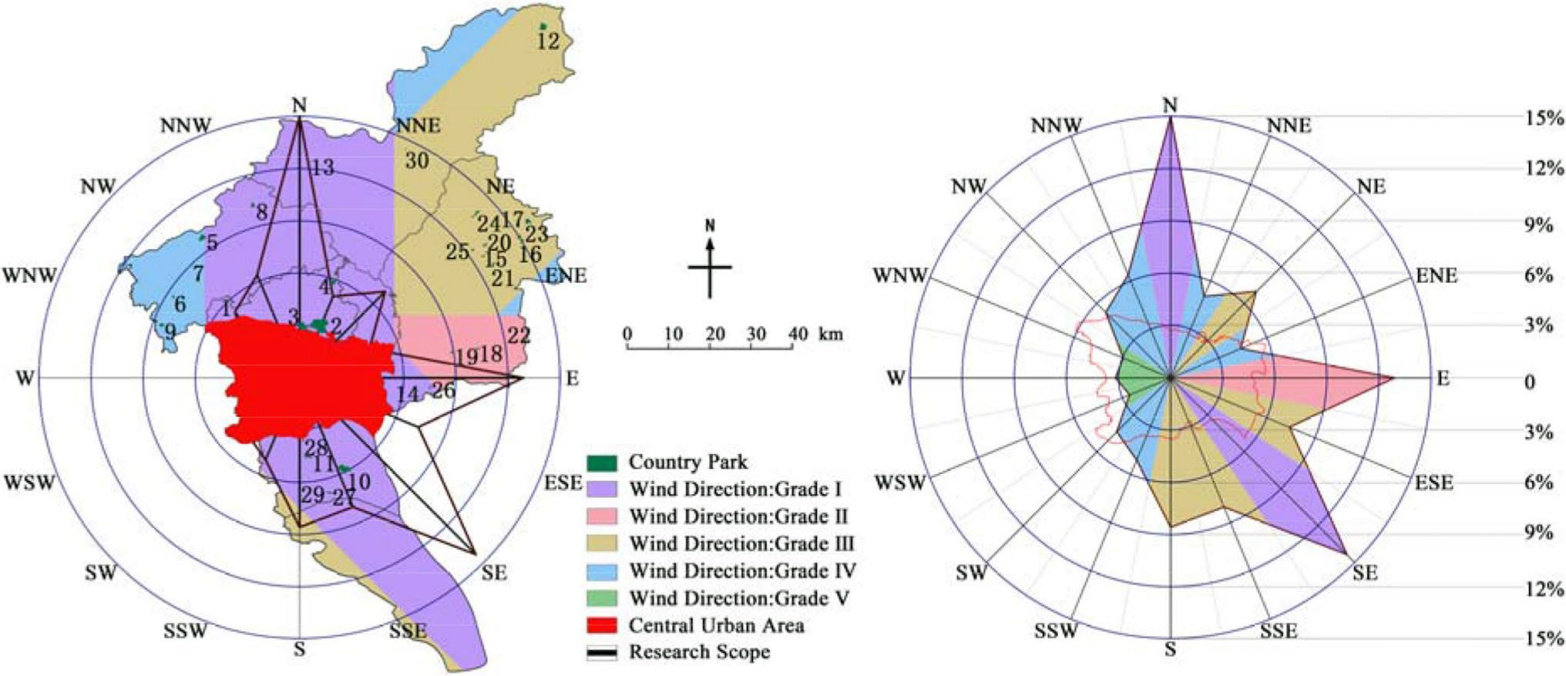
Wind direction of the country parks

According to the statistics of wind direction (Table 10 and Fig. 8), there were 13 parks in grade I area, 3 in grade II area, 10 in grade III area, 4 in grade IV area, and none in grade V area. The results showed that the wind direction of country parks was an unfavorable factor as a whole. The dominant wind direction in Guangzhou was significant, with northwest wind prevailing in winter and southeast wind prevailing in summer (Chen et al. 2021). It is worth noting that the wind belt has a certain width on the map, which is consistent with the width of the corresponding angle of the urban. Therefore, the wind zones of 16 directions had certain overlapping regions, and the parks in the overlaps followed the principle of strict.

**Table 10:** Assignment results of the wind direction

Park number	1	2	3	4	5	6	7	8	9	10	11	12	13	14	15
Wind direction assignment	I	I	I	I	IV	IV	IV	I	IV	I	I	III	I	I	III
Park number	16	17	18	19	20	21	22	23	24	25	26	27	28	29	30
Wind direction assignment	III	III	II	II	III	III	II	III	III	III	I	I	I	I	III

### Distance from the city center evaluation

**Fig. 9:**
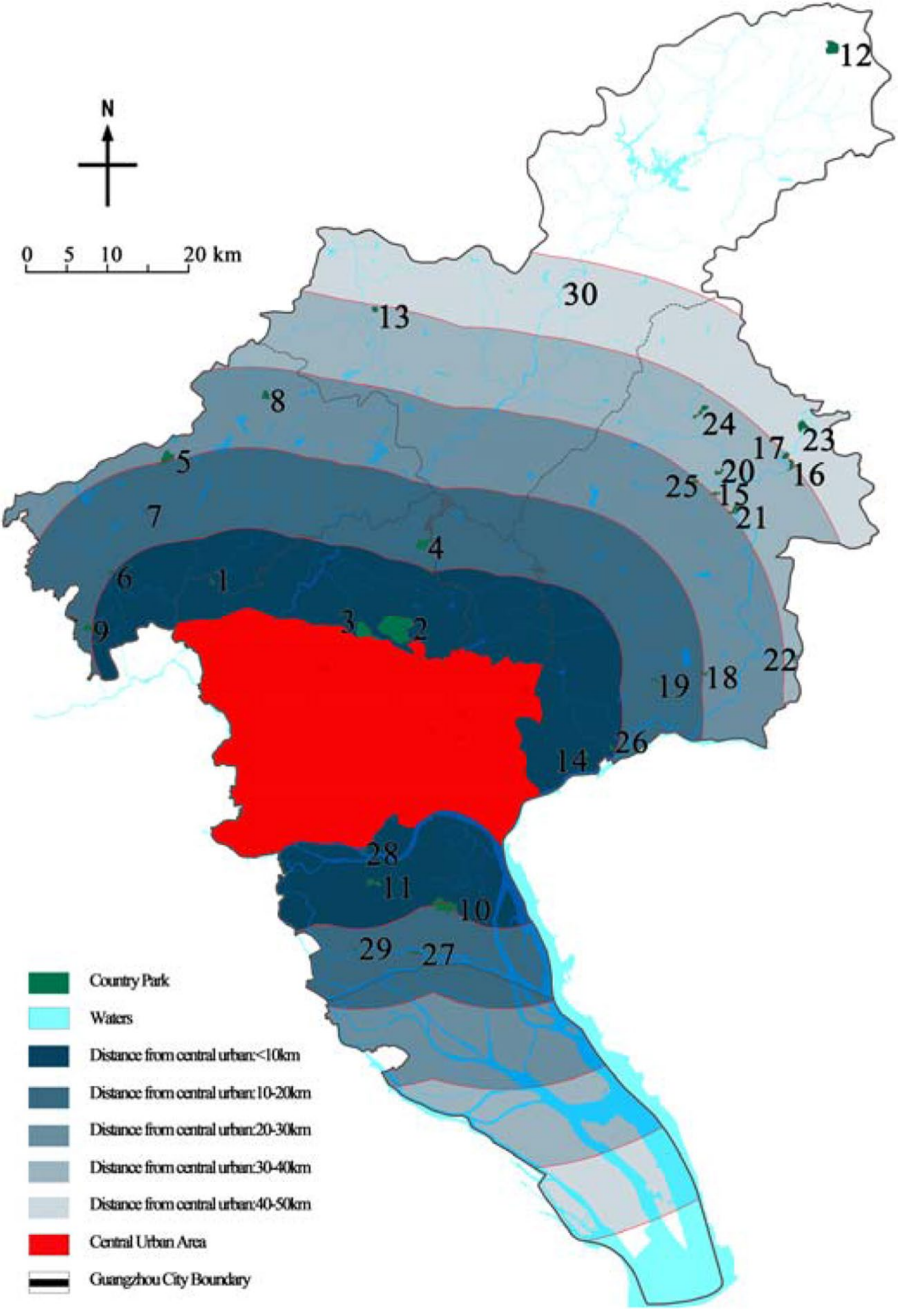
Distance from urban of the country parks

According to the statistics of distance from the city center (Table 11 and Fig. 9), there were 8 parks in grade I area, 8 in grade II area, 3 in grade III area, 8 in grade IV area, and 3 in grade V area. Among them, more than 50% of the parks were located in grade I and II areas, which follow the principle of selecting the nearest location for the service, but it is not an ideal place for emergency medical facilities. Because when the park is used as a construction site, a safety isolation area no less than 20 m should be set up, and corresponding isolation measures should be taken in advance.

**Table 11:** Assignment results of the distance from the city center

Park number	1	2	3	4	5	6	7	8	9	10	11	12	13	14	15
Distance from the city center assignment	I	I	I	II	III	II	II	III	II	I	I	V	IV	I	IV
Park number	16	17	18	19	20	21	22	23	24	25	26	27	28	29	30
Distance from the city center assignment	IV	IV	II	II	IV	IV	IV	V	IV	III	I	II	I	II	V

### Impermeability evaluation

**Fig. 10:**
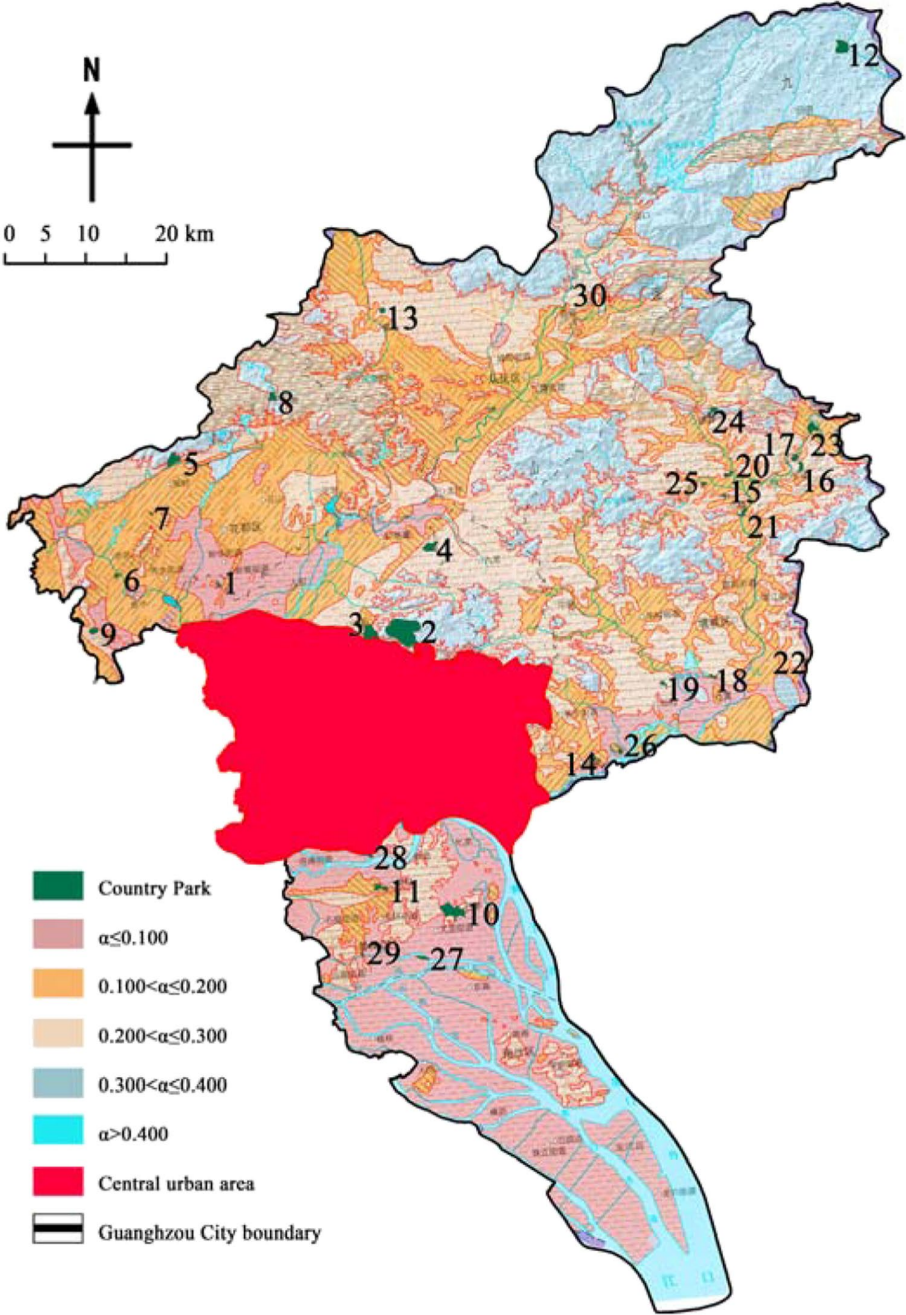
Impermeability of the country parks

According to the statistics of impermeability (Table 12 and Fig. 10), there were 3 parks in grade I area, 2 in grade II area, 13 in grade III area, 8 in grade IV area, and 2 in grade V area. It should be noted that the lithology of grade I and II area was quaternary alluvial diluvial gravel sand with poor water resistance. If emergency medical facilities are built in these parks, the anti-seepage treatment under the site should be strengthened according to the standards of landfill site (Dai et al. 2013).

**Table 12:** Assignment results of the impermeability

Park number	1	2	3	4	5	6	7	8	9	10	11	12	13	14	15
Impermeability assignment	V	II	III	III	II	III	IV	III	IV	III	IV	II	III	IV	III
Park number	16	17	18	19	20	21	22	23	24	25	26	27	28	29	30
Impermeability assignment	III	III	V	III	III	IV	III	IV	II	IV	IV	I	I	I	III

### Transport duration evaluation

**Fig. 11:**
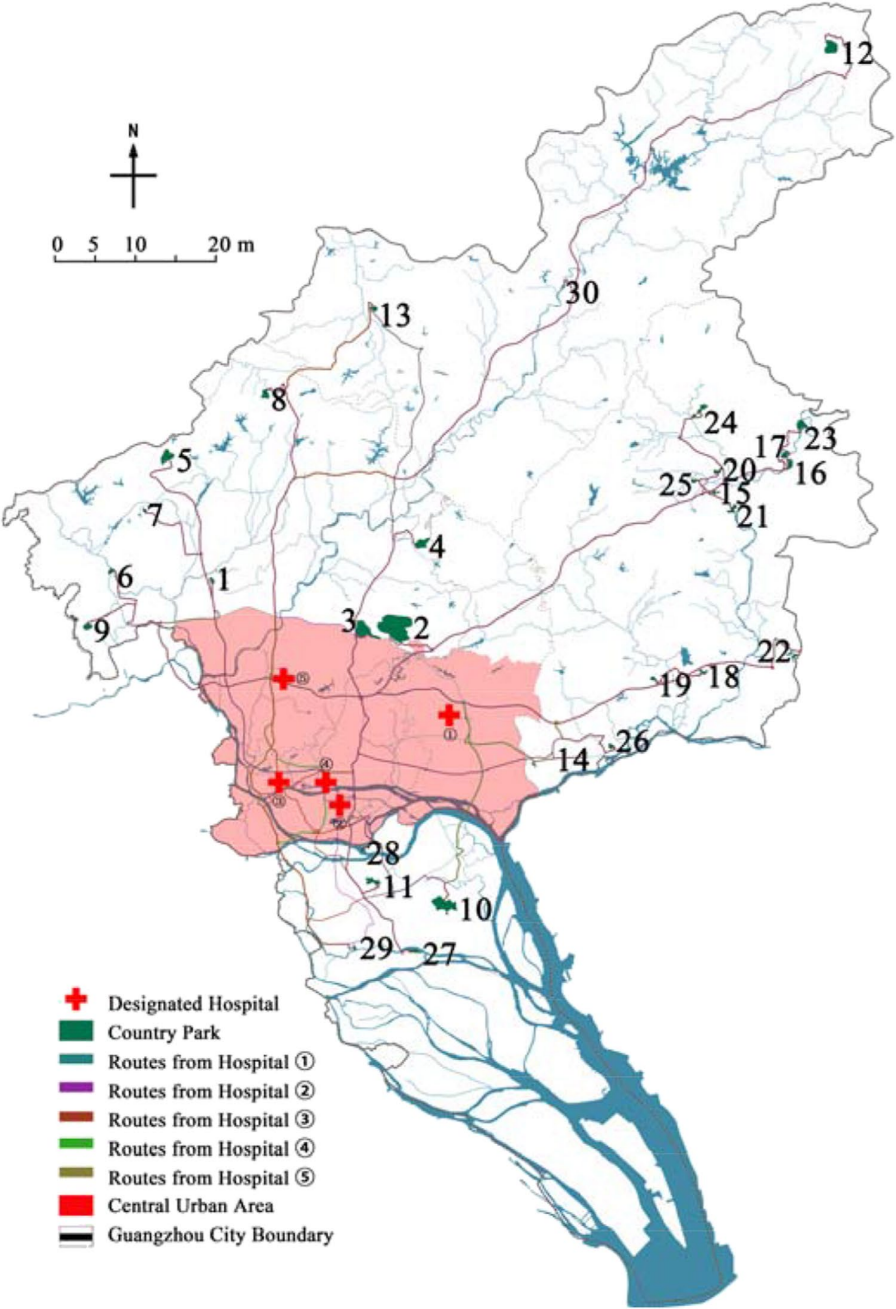
Transport duration of the country parks

According to the statistics of transport duration (Table 13 and Fig. 11), there were 2 parks in grade I area, 3 in grade II area, 16 in grade III area, 7 in grade IV area, and 2 in grade V area. More than 80% of the parks were located in areas above grade III, and the transport duration was less than 100 minutes. As a whole, the transport duration is a favorable factor for site selection. It should be pointed out that the traffic time was not completely proportional to the traffic distance. For example, there are many intersections around the country parks near the central urban, so the advantage of traffic duration was not prominent.

**Table 13:** Assignment results of the transport duration

Park number	1	2	3	4	5	6	7	8	9	10	11	12	13	14	15
Transport duration assignment	II	V	V	IV	I	III	I	III	II	III	IV	II	IV	III	IV
Park number	16	17	18	19	20	21	22	23	24	25	26	27	28	29	30
Transport duration assignment	III	III	III	IV	III	III	III	III	III	IV	III	III	IV	III	III

## Results

**Fig. 12:**
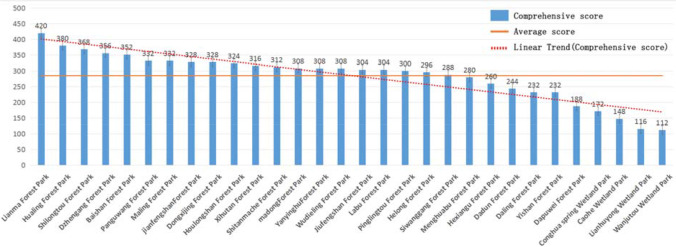
Comprehensive score of the country parks

According to the evaluation results of the eight impact factors, the comprehensive scores of 30 country parks were calculated through Eq. 4, and the results are shown as Fig. 12.

Among the 30 country parks, Lianma Forest Park had the highest comprehensive score. It is a forest and mountain type, with high green coverage, good forest enclosure, and good ecological environment, which could provide patients with a better healthcare environment. It also had the available construction area of 215.7 hm^2^, high integration, and is far away from the waters, which could provide safe and sufficient space for emergency medical facilities. It is worth noting that the geological water separation performance of Lianma Forest Park is poor, so the anti-seepage treatment under the site should be strengthened with reference to the landfill standard. In addition, Hualing Forest Park, Shilongtou Forest Park, Dazhengang Forest Park, Baishan Forest Park, and other comprehensive conditions are also superior, which can be included in the emergency plan as an alternative.

## Discussion

By analyzing the research results, the following can be concluded:

(1) The highest score was 420 and the lowest score was 112 out of 500, and the former was 3.75 times higher than the latter, indicating that this method can significantly distinguish the advantages and disadvantages of the parks.

(2) The average score of country parks was 285, which was medium to low. Among them, there were 10 parks with a score of less than 285, 3 parks with a score of 285–300, 12 parks with a score of 300–350, 4 parks with a score of 350–400, and 1 park with a score of more than 400, indicating that the method has obvious hierarchical differentiation.

(3) Among the top ranked parks, the park type, available construction area, spatial fragmentation degree, and distance from waters have outstanding advantages. The top 5 country parks are mainly forest type, with high site integration, available construction area greater than 70 hm^2^, and distance from waters greater than 1 km. This result was highly consistent with the weight assignment of each impact factor.

(4) The advantages of wind direction and water-resisting property are not obvious, and their grade II both appeared in the top 5 country parks. The two factors are largely limited by established conditions and can be solved or alleviated by means of green belt and impervious layer.

This article takes the disaster prevention and risk avoidance function of urban green space as the starting point to explore the connection between urban green space and public health events. The difference is that the so-called “disaster prevention and risk aversion” in the past mainly refers to the emergency measures taken in response to disasters such as earthquakes, fires, tsunamis, and floods. These disasters often only occur in a certain region or country, and their impact scope and severity are far less than the COVID-19. Therefore, should we try to consider turning individual types of urban green spaces into green public spaces with specific functions? As described in the article, utilizing the high degree of compatibility between country parks and emergency medical facility site selection, following the principle of “Disaster Prevention and Reduction,” country parks are included in the emergency plan for public health emergencies, alleviating the difficulties of scientific site selection and timely construction of emergency medical facilities. In addition, country parks can also provide places for medical staff and patients in rehabilitation to recover their physical and mental health. The COVID-19 is not the first public health emergency in human history, nor will it be the last. Therefore, establishing a connection between urban green spaces and public health emergencies can provide a strong and sustainable public health emergency response mechanism, which can better respond to similar emergencies. The disaster prevention and risk avoidance function of country parks needs to shift towards public health and safety response, fully play its role in sudden public health events, improve the basic level of urban public health, and make contributions to the health and safety of the city.

The country park selected as the target of emergency medical facilities should be upgraded and transformed according to the construction standard of infectious disease emergency medical facilities. (1) In order to meet the needs of medical rescue work, it is necessary to renovate the park site, provide a suitable site for setting up tents or emergency medical facilities, and ensure that the site is level, easy to use, and clean. (2) Establish necessary infrastructure within the country park, such as water, electricity, natural gas, and ventilation, to ensure the normal operation of the building’s internal pipeline system. (3) Strengthen and enhance the functionality and coverage of required communication equipment, including emergency broadcasting, radio, and network communication. Ensure timely access to external information and guidance. (4) Upgrade the sewage treatment system of the rural park and equip it with treatment equipment that meets the “Technical Specification for Centralized Disposal of Medical Waste” to treat various types of garbage and wastewater. (5) Choose plants that can absorb harmful substances and have health functions to form a favorable microclimate and provide patients with a good rehabilitation environment. (6) In conjunction with the management agencies of the park, develop contingency plans for public health events and establish emergency plans and corresponding emergency procedures to ensure timely use.

## Conclusion

By analyzing the characteristics of public health event outbreak and transmission and combining the basic conditions required for emergency medical facilities, this study identified 8 influencing factors for using country parks as emergency medical facility sites and conducted an evaluation study using 30 country parks in Guangzhou City as examples. The results of the study confirmed that country parks can be used as sites for emergency medical facilities. The evaluation used the Delphi method combined with the AHP method, combining qualitative and quantitative, and further quantitative analysis of each index while fully reflecting the experts’ opinions, but there are still some subjective judgment and perceptual cognitive factors, and the subsequent research can further expand and optimize the evaluation factors to make the qualitative and quantitative more closely integrated and establish a more comprehensive and complete evaluation system. Due to the large differences in conditions in different countries and regions, the degree of influence of each influencing factor on the siting of emergency medical facilities varies. Therefore, researchers should increase or decrease the types of influencing factors according to the situation of the study site and adjust the weights according to the degree of influence in order to bring into play the favorable conditions of the target area and avoid the unfavorable factors. In addition, this study lacks consideration of the construction cost of emergency medical facilities, procurement cost of emergency resources, and transportation cost, and the next study will be based on this, considering the minimization of comprehensive costs, optimal siting of emergency facilities, and rational planning of the number of allocated medical resources and the path of transferring infected patients.

## Data Availability

The datasets used and analyzed during the current study are available from the corresponding author on reasonable request.
